# Identification and characterization of a hyperthermophilic GH9 cellulase from the Arctic Mid-Ocean Ridge vent field

**DOI:** 10.1371/journal.pone.0222216

**Published:** 2019-09-06

**Authors:** Anton A. Stepnov, Lasse Fredriksen, Ida H. Steen, Runar Stokke, Vincent G. H. Eijsink

**Affiliations:** 1 Faculty of Chemistry, Biotechnology and Food Science, NMBU—Norwegian University of Life Sciences, Ås, Norway; 2 Department of Biological Sciences and KG Jebsen Centre for Deep Sea Research, University of Bergen, Bergen, Norway; Institut National de la Recherche Agronomique, FRANCE

## Abstract

A novel GH9 cellulase (AMOR_GH9A) was discovered by sequence-based mining of a unique metagenomic dataset collected at the Jan Mayen hydrothermal vent field. AMOR_GH9A comprises a signal peptide, a catalytic domain and a CBM3 cellulose-binding module. AMOR_GH9A is an exceptionally stable enzyme with a temperature optimum around 100°C and an apparent melting temperature of 105°C. The novel cellulase retains 64% of its activity after 4 hours of incubation at 95°C. The closest characterized homolog of AMOR_GH9A is *Tf*Cel9A, a processive endocellulase from the model thermophilic bacterium *Thermobifida fusca* (64.2% sequence identity). Direct comparison of AMOR_GH9A and *Tf*Cel9A revealed that AMOR_GH9A possesses higher activity on soluble and amorphous substrates (phosphoric acid swollen cellulose, konjac glucomannan) and has an ability to hydrolyse xylan that is lacking in *Tf*Cel9A.

## Introduction

Cellulose is a main structural component of plant biomass and the most abundant carbohydrate on Earth. It is composed of repeating d-anhydroglucose units linked by β(1→4) glycoside bonds [1,2]. Individual cellulose chains are arranged into crystalline microfibrils that are stabilized by an extensive network of intra- and intermolecular hydrogen bonds [1]. The renewability of cellulose makes it an attractive source of green energy, but its exploitation is complicated by its resistance to depolymerization [3].

In Nature, degradation of cellulose is carried out by the synergistic action of endo-acting and exo-acting enzymes that include glycosyl hydrolases (GHs) and lytic polysaccharide monooxygenases (LPMOs) [4,5]. However, despite decades of research, industrial enzymatic processing of cellulosic plant biomass is still hampered by enzyme costs [6]. Thus, there is a clear incentive for discovering better cellulases.

The temperature optima of cellulases in currently available commercial enzyme cocktails are typically around 50°C [7], i.e., not particularly high and likely not optimal, for example considering the risk of microbial contamination. The introduction of thermostable enzymes could be beneficial since this would allow the use of higher temperatures, resulting in increased substrate solubility, lower viscosity and reduced microbial growth [8]. Furthermore, the use of thermostable cellulases can simplify process design by minimizing or eliminating cooling periods between stages that require different temperatures (e.g. between heat pre-treatment and enzymatic conversion) [8, 9].

Metagenomics has proven to be a powerful tool for the discovery of thermostable enzymes from microbial sources. The crucial advantage of this approach is the ability to access extremophile genomes in a culture-independent manner [10]. Metagenomics has been successfully used to mine for novel enzymes in various high temperature environments such as compost, hot springs, deserts and deep sea vent fields [11]. Deep sea vents are promising niches for the search of extremozymes because they accommodate an impressive variety of microorganisms some of which can grow at temperatures as high as 121°C [12]. Although deep-sea hydrothermal vents are characterized by lack of plant biomass [13] cellulolytic activity is not uncommon in the bacterial communities in these environments [14, 15]. Microbial biofilms are thought to be the most likely source of complex polysaccharide substrates around hydrothermal vents [16].

In recent years, we have been exploring the biodiversity of the Jan Mayen hydrothermal vent field at the Arctic Mid-Ocean Ridge, where temperatures can rise up to 260°C [17]. In this paper, we report on a novel hyperthermophilic GH9 cellulase, AMOR_GH9A, which was discovered by *in silico* mining of a metagenome from the Jan Mayen hydrothermal vent field. The closest characterized homolog of AMOR_GH9A is *Tf*Cel9A, a processive endocellulase from the moderately thermophilic bacterium *Thermobifida fusca* [18]. We have also expressed and purified *Tf*Cel9A, which was then used as a reference enzyme in a comparative assessment of AMOR_GH9A functional properties. The results show that AMOR_GH9A has higher thermal stability and broader substrate specificity than its homolog from *T*. *fusca*.

## Materials and methods

### Sample collection, sequencing and identification of genes

A sample of unbleached Norway spruce (*Picea abies*) that had been pretreated by sulfite-pulping using the BALI process [19, 20], at Borregaard AS (Sarpsborg, Norway), was incubated for one year in ~70°C hot sediments at the Arctic Mid-Ocean Ridge (AMOR), 570 m below sea level, and then recovered by a remotely operated vehicle. In short, 1 g of spruce material was mixed with approximately 16 mL of sediment sampled at the site and placed in the bottom chamber of a titanium incubator (2.5 cm chamber length, 16 mL chamber volume, 1 mm pores). The sampling was performed in a responsible way in accordance with the Norwegian Marine Resource Act and did not involve endangered or protected species. No permits were required to access the sampling site. DNA was extracted from 6.9 g of material and 1.1 μg of DNA was submitted for sequencing. The sampling procedure and the methods used for DNA extraction and sequencing have been described in detail elsewhere [21, 22]. Filtering and assembly of the raw Illumina MiSeq 300 paired-end reads were performed using the CLC genomics workbench utility (Qiagen, v.9.5.3) as previously described in [21, 22]. Open reading frames were predicted using Prodigal software [23, 24]. The resulting metagenomic dataset was mined for putative glycosyl hydrolases using the dbCAN service (csbl.bmb.uga.edu/dbCAN) [25]. The signal peptides of the candidate genes were annotated using SignalP [26]. The full characteristics of the metagenomic dataset will be published elsewhere.

The sequence-based mining led to the identification of a 2065 bp gene encoding a putative GH9 cellulase (AMOR_GH9A). The NCBI BLAST server (https://blast.ncbi.nlm.nih.gov/Blast.cgi) was used to identify homologues of AMOR_GH9A. The sequence of AMOR_GH9A has been submitted to Genbank under accession number MK869727, and the DNA sequence of *Tf*Cel9A was obtained from GenBank (accession number L20093.1).

### Gene synthesis and subcloning

The AMOR_GH9A and *Tf*Cel9A genes were codon optimized for expression in *E*. *coli* and synthesised by GenScript (Piscataway, NJ, USA). The genes were then amplified by PCR using Q5 high-fidelity DNA polymerase (New England Biolabs, Ipswich, MA, USA). The forward and reverse PCR primers incorporated plasmid-specific regions for ligation-independent cloning [27] to the pNIC-CH expression vector (AddGene, Cambridge, MA, USA) (see S1 Table for details). The PCR products were purified from 1% agarose gels using a Nucleospin Gel Clean-Up kit (Macherey-Nagel, Düren, Germany). After ligation-independent cloning, the reaction mixture was used for heat-shock transformation of OneShot TOP10 *E*. *coli* competent cells (Invitrogen, Carlsbad, USA) as recommended by the supplier. The transformed cells were incubated in SOC medium for 60 minutes at 37°C prior to plating on LB agar medium supplied with 50 μg/ml kanamycin and 5% (w/v) sucrose. The clones from overnight incubation at 37°C were screened for the target inserts by colony PCR using RedTaq polymerase (VWR International, Radnor, PA, USA) and pNIC-CH forward and reverse sequencing primers (see S1 Table). Positive clones were transferred to liquid LB medium with 50 μg/ml kanamycin for overnight cultivation at 37°C, 200 rpm. The pNIC-CH plasmids harbouring target genes were purified using a NucleoSpin Plasmid kit (Macherey-Nagel, Düren, Germany) and the sequence of these expression vectors was confirmed by Sanger sequencing (GATC, Konstanz, Germany). The resulting expression plasmids code for AMOR_GH9A or *Tf*Cel9A without a signal peptide, starting with a methionine residue introduced at the N-terminus of the mature protein, and with a C-terminal affinity tag (“-AHHHHHH”).

### Expression and purification

AMOR_GH9A and *Tf*Cel9A expression strains were established through transformation of the expression plasmids to competent *E*. *coli* BL-21 Star^TM^ (DE3) cells (Invitrogen, Carlsbad, USA) according to the supplier’s protocol. The transformed cells were incubated in LB medium at 37°C for 1 hour prior to plating on LB agar medium with 50 μg/ml kanamycin, followed by overnight cultivation at the same temperature. The resulting clones were transferred to 500 ml of Terrific Broth (TB) medium with 50 μg/ml kanamycin and cultivated for 24 hours in a Harbinger system (Harbinger Biotechnology & Engineering, Markham, Canada) at 23°C. The cultures were then induced by adding ITPG to a final concentration of 1 mM, and incubated for another 24h at 23 ^o^C. The cells were harvested by centrifugation at 5000 x g for 15 minutes at 4°C, using a Beckman Coulter centrifuge (Brea, CA, USA) and resuspended in 50 ml 50 mM Tris-HCl buffer pH 8.0 containing 500 mM NaCl and 5 mM imidazole. The cell suspensions were subjected to sonication on ice using a Vibracell sonicator (Sonics & Materials Inc., Newtown, Connecticut, USA) with 5 seconds on/off pulses for 3 minutes at 30% amplitude. The debris was removed by centrifugation at 15,000 x *g* for 15 minutes at 4°C and the supernatant was filtered through a 0.22 μm syringe filter (Sarstedt, Nümbrecht, Germany), yielding sterile cell-free extracts, which were stored at 4°C prior to enzyme purification.

AMOR_GH9A and *Tf*Cel9A proteins were isolated from cell-free extracts using metal affinity chromatography on a Ni^2+^ affinity HisTrap^TM^ HP 5 ml column (GE Healthcare, Chicago, USA). The enzymes were eluted with a linear gradient of imidazole (5–500 mM) in 50 mM Tris-HCl buffer, pH 8.0, containing 500 mM NaCl. Chromatography fractions were analyzed by SDS-PAGE (Bio-Rad, Hercules, California, USA). Fractions containing purified enzymes were pooled and concentrated using 10,000 MWCO Vivaspin ultrafiltration tubes (Sartorius, Göttingen, Germany), with concomitant buffer exchange to 50 mM Tris-HCl, pH 8.0, containing 200 mM NaCl. The enzyme concentrations were determined by measuring optical absorbance at 280 nm with a Biophotometer UV-VIS spectrophotometer (Eppendorf, Hamburg, Germany), using theoretical extinction coefficients (web.expasy.org/protparam). The protein stock solutions were stored at 4°C.

### Optimal operating conditions

The temperature optima of the enzymes were assessed by incubation of 1μM AMOR_GH9A or 2 μM *Tf*Cel9A with 1% (w/v) carboxymethyl cellulose (CMC) for 6 minutes at temperatures ranging from 20°C to 100°C in citrate-phosphate buffer pH 5.7 (AMOR_GH9A) or pH 6.2 (*Tf*Cel9A). The pH optima were determined by carrying out the same reactions in various citrate-phosphate (pH 3.0–7.6) and glycine-NaOH buffers (pH 9.3–10.8) at 98°C (AMOR_GH9A) or 65°C (*Tf*Cel9A). The pH of the buffer solutions was set at room temperature. The experiments were conducted in a thermomixer (Eppendorf, Hamburg, Germany) at 600 rpm. The cellulase activity was determined by measuring the release of reducing sugars using the 3,5-dinitrosalicylic acid (DNS) reagent [28] and glucose as a standard.

The effect of salt on the performance of AMOR_GH9A was determined by incubation of 1 μM enzyme and 1% (w/v) CMC in citrate-phosphate buffer pH 5.7 at 98°C with 0, 100, 500, 1000 or 2000 mM NaCl. Product formation was analyzed using the DNS assay, as described above.

### Thermal stability

The thermal stability of AMOR_GH9A and *Tf*Cel9A was assessed by measuring the residual activity of the enzymes on 1% (w/v) CMC after up to 24 hours of pre-incubation in citrate-phosphate buffer pH 5.7 (AMOR_GH9A) or pH 6.2 (*Tf*Cel9A). The pre-incubation was performed at 98, 90, 85, and 80°C (AMOR_GH9A) or at 65, 60, 55 and 50°C (*Tf*Cel9A). The reactions with CMC were carried out for 6 minutes at 98°C (AMOR_GH9A) or 65°C (*Tf*Cel9A) and product formation was quantified with the DNS assay as described above.

### Apparent melting temperature

Differential scanning calorimetry (Nano-Differential Scanning Calorimeter III, Calorimetry Sciences Corporation, Lindon, USA) was used to determine the apparent melting temperatures of AMOR_GH9A and *Tf*Cel9A. The protein solutions were desalted using MiniTrap^tm^ G-25 gel filtration columns and citrate-phosphate running buffers with pH 5.7 (AMOR_GH9A) or pH 6.2 (*Tf*Cel9A). These running buffers were utilized as reference samples in the subsequent calorimetry experiments. The protein solutions (final protein concentration approximately 0.5 mg/ml) and the reference solutions were filtered through a 0.22 μm syringe filter (Sarstedt, Nümbrecht, Germany) and degassed for 5 minutes using a ThermoVac system (GE Healthcare, Chicago, IL, USA) prior to data collection. The calorimetry was carried out in a pressurized chamber (4 atm) at 20–130°C temperature range with 1°C/min scan rate. The data were processed using the NanoAnalyze software provided by TA Instruments (New Castle, DE, USA). The buffer baselines were subtracted from the enzyme melting curves.

### Substrate specificity

Avicel PH-101 (Sigma-Aldrich, St. Louis, MO, USA) was selected as a model crystalline substrate in this study. Phosphoric-acid swollen cellulose (PASC) was prepared from Avicel as described in [29]. Beechwood xylan and konjac glucomannan were purchased from Megazyme (Wicklow, Ireland) and prepared according to the supplier protocol. The reactions were carried out in citrate-phosphate buffer pH 5.7 at 85°C (AMOR_GH9A) or in citrate-phosphate buffer pH 6.2 at 55°C (*Tf*Cel9A) with 1 μM enzyme. The substrate concentrations were 1% (w/v) for Avicel and 0.5% (w/v) for the other substrates. Aliquots were taken at various time points and the reactions were stopped by addition of NaOH to 100 mM final concentration. The products were quantified using the DNS assay and glucose standards.

### Product analysis by HPAEC-PAD

Degradation products from cellulose and xylan were analyzed using high-performance anion-exchange chromatography with pulsed amperometric detection (HPAEC-PAD). The cellooligosaccharides were separated using a Dionex ICS3000 system (Thermo Scientific, San Jose, CA, USA) equipped with a CarboPac PA1 2 × 250 mm analytical column. A stepwise gradient with an increasing amount of eluent B (eluent B is 0.1 M NaOH and 1 M NaOAc; eluent A is 0.1 M NaOH) was applied starting right after sample injection, as follows: 0–10% B over 10 min, 10–30% B over 25 min, 30–100% B over 5 min, 100–0% B over 1 min, 0% B over 9 min. Data analysis was performed using Chromeleon 7.0 software. Cellooligosaccharide standards with a degree of polymerization of one to five (DP1—DP5) and xylo-oligosaccharide standards with a degree of polymerization of one to six (DP1 –DP6) were purchased from Megazyme (Wicklow, Ireland) and used to identify the products.

### Product analysis by MALDI-TOF MS

The products of xylan degradation were identified using a matrix-assisted laser desorption/ionization time-of-flight (MALDI-TOF) UltrafleXtreme mass spectrometer (Bruker Daltonics GmbH, Bremen, Germany) equipped with a Nitrogen 337-nm laser. 1 μl of reaction mixture was added to 2 μl of 9 mg/ml 2,5-dihydrooxybenzoic acid (DHB) solution on a MTP 384 ground steel target plate (Bruker Daltonics). After air-drying, spectral data was acquired and processed using Bruker flexControl and flexAnalysis software.

## Results and discussion

### Metagenomic data analysis

**Fig 1:**
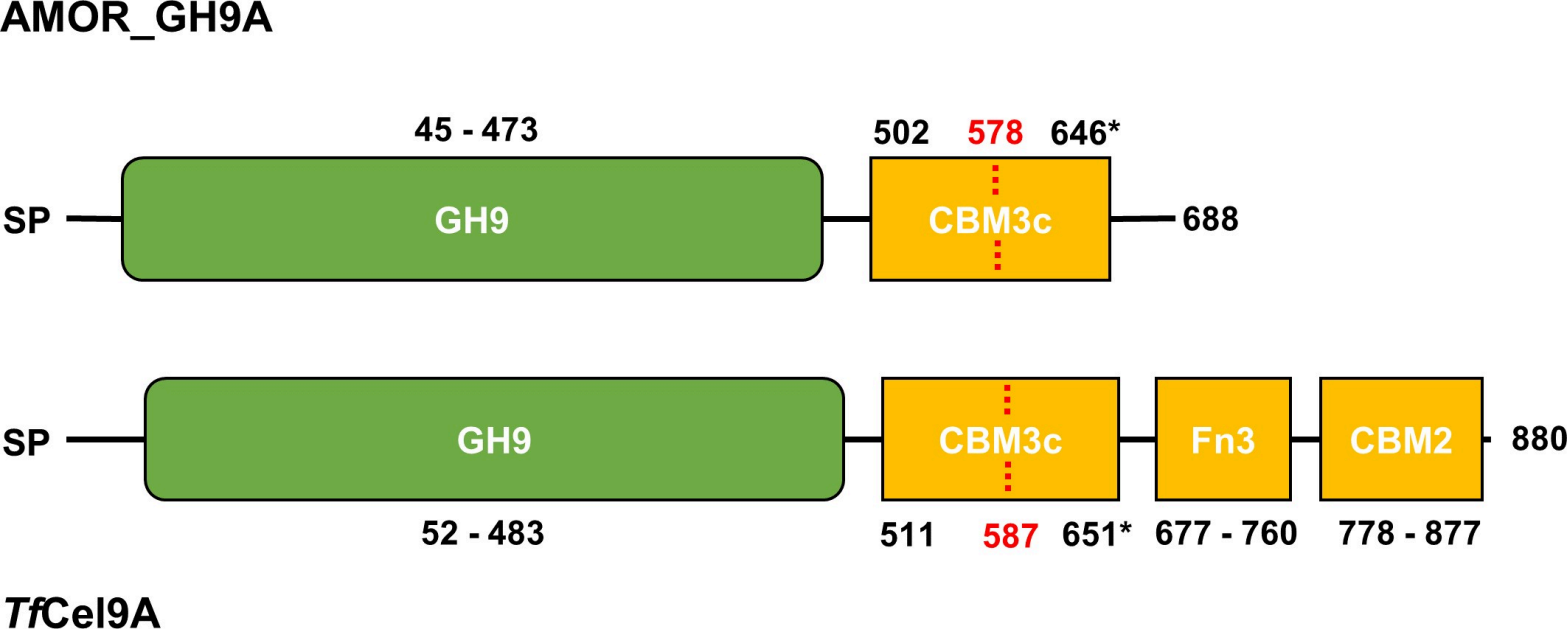
Domain architecture of AMOR_GH9A and *Tf*Cel9A cellulases. SP, signal peptide; GH9, catalytic domain; CBM2/CBM3c, family 2/family 3c cellulose-binding module; Fn3, type III fibronectin domain. The dotted line and number in red colour indicate the boundaries of the CBM3c domain according to the erroneous Pfam prediction. The domain coordinates marked with “*” were derived from sequence comparisons and the crystal structure of *Tf*Cel9A. See text for details.

After sequencing and assembly of metagenomic data, a 2065 bp gene encoding a putative GH9 cellulase was identified using the dbCAN annotation tool. The candidate enzyme was named AMOR_GH9A. According to the Pfam domain classification server [30], AMOR_GH9A is a 688 residue protein (Fig 1) comprising a signal peptide, a catalytic GH9 domain and a CBM3 cellulose binding module.

BLAST searches identified a hypothetical endoglucanase from the thermophilic marine bacterium *Ardenticatena maritima* as having the highest degree of sequence similarity to AMOR_GH9A (77.8% identity between catalytic domains, sequence ID: WP_060687350.1). *Ardenticatena maritima* was isolated from a hydrothermal field sediment and can grow at temperatures as high as 75° C [31]. The closest characterized homolog of AMOR_GH9A is *Tf*Cel9A from the cellulolytic model actinomycete *Thermobifida fusca (*67.7% identity between catalytic domains). *Tf*Cel9A is a well-known thermostable GH9 cellulase with a complex domain architecture and an endo-processive mode of action [32, 33]. *Tf*Cel9A consists of a signal peptide, an N-terminal catalytic domain, two cellulose binding modules and a fibronectin type III domain (Fig 1). In this study, *Tf*Cel9A was selected as the reference enzyme to assess AMOR_GH9A thermostability and substrate specificity via direct comparison.

**Fig 2:**
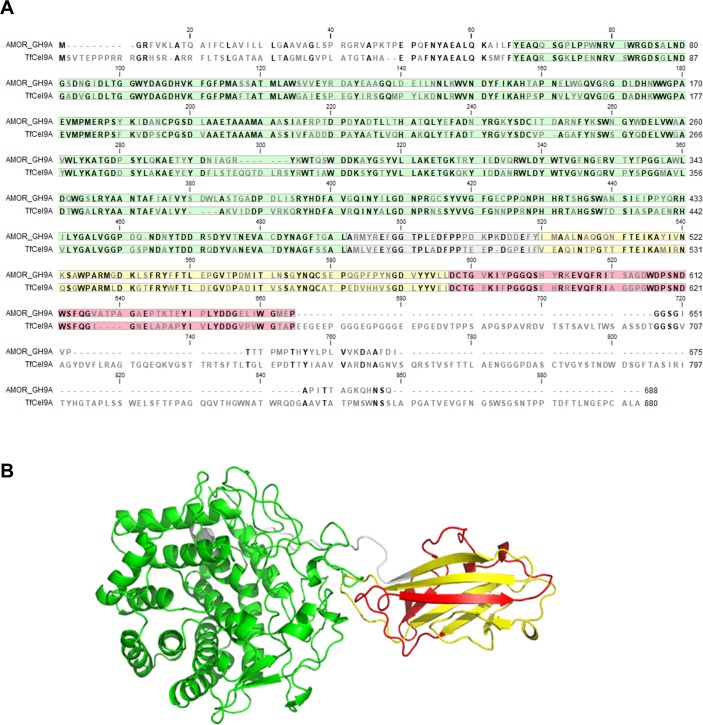
AMOR_GH9A compared to *Tf*Cel9A. Panel (A) shows a sequence alignment of AMOR_GH9A and *Tf*Cel9A, whereas panel B shows the crystal structure of a fragment of *Tf*Cel9A comprising the catalytic domain and the CBM3c domain (residues 47–651) that was obtained by limited proteolysis [34]. The protein regions are marked with colour as follows: green, catalytic domain; grey, linker; yellow, N-terminal part of the CBM3c that is recognized by Pfam; red, C-terminal part of the CBM3c that is not recognized by Pfam (see text for details). The conserved amino acid residues are indicated by bold font. Note that AMOR_GH9A is shorter than *Tf*Cel9A and that the alignment of the C-terminal “tail” of AMOR_GH9A (residues 665–688) with the much longer C-terminal part of *Tf*Cel9A is inaccurate and does not necessarily indicate structural or functional similarities.

Of note, both Pfam and dbCAN did not predict the domain boundaries of the CBM3 in AMOR_GH9A correctly, recognizing only the N-terminal half of this domain (Fig 1). Sequence alignment with *Tf*Cel9A (Fig 2A) and the X-ray structure of *Tf*Cel9A (Fig 2B; [34]) clearly show that the CBM3 comprises approximately 140 residues, as one would expect [35]. Domain annotation of *Tf*Cel9A with Pfam gave a similarly incorrect result. The CBM3 domain of AMOR_GH9A lacks the so-called “planar strip” (a conserved array of mostly aromatic amino acids involved in binding to crystalline substrates) and belongs to subfamily CBM3c [32, 35].

### Protein production

Genes encoding AMOR_GH9A and *Tf*Cel9A were codon optimized for expression in *E*. *coli*, synthesized and then cloned into pNIC-CH vectors using ligation-independent cloning. The enzymes were produced (S1 Fig) in *E*. *coli* BL-21 Star^TM^ (DE3), without signal peptides and with a C-terminal affinity tag for purification by metal affinity chromatography. AMOR_GH9A and *Tf*Cel9A were produced in soluble form and the final yield was approximately 90 mg of purified protein per liter of *E*. *coli* culture for both enzymes.

### Optimal operating conditions

**Fig 3:**
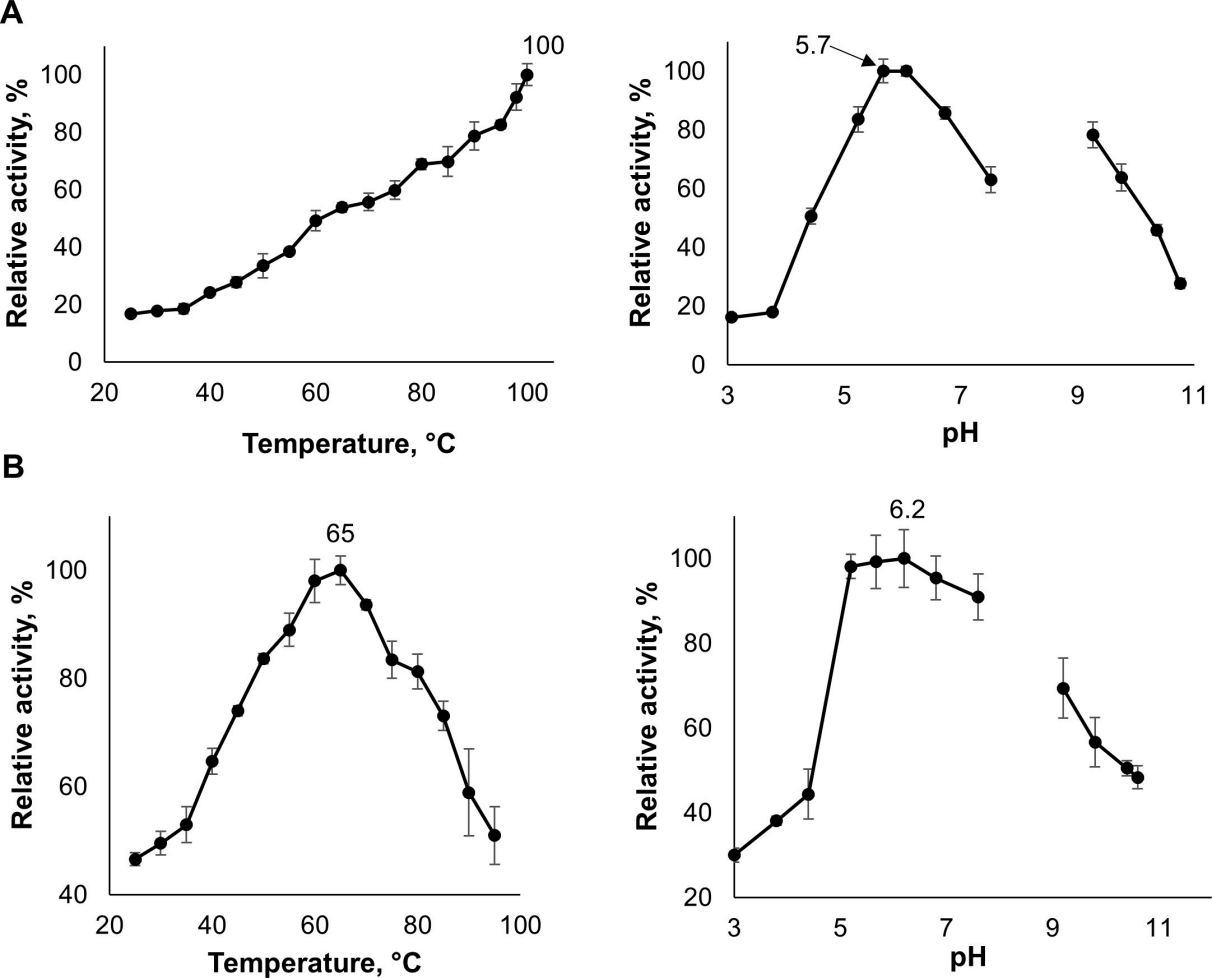
Optimal conditions for AMOR_GH9A and *Tf*Cel9A. Panels (A) and (B) show the temperature and pH optima of AMOR_GH9A (A) and *Tf*Cel9A (B). Temperature optima were determined at pH 5.7 and 6.2 and pH optima were determined at 98 ^o^C and 65 ^o^C, for AMOR_GH9A and *Tf*Cel9A, respectively. Enzyme activities were assessed by measuring product formation from CMC after 6 min reactions. The maximum level of product formation was set to 100% and the temperatures or pH values at which this maximum level was obtained are shown in the graph. Note that two different buffers were used in the determination of the pH optimum, citrate-phosphate, covering pH 3.0–7.6 and glycine-NaOH, covering pH 9.2–10.7. The pH values displayed in the figure were measured at room temperature. While the temperature dependency of the pH of the citrate-phosphate buffer is close to negligible [36], the temperature dependency of the pH of the glycine-NaOH buffer is considerable (*d*p*K*a_2_/*dt* = -0.025, [37]). Thus, considering the assay temperatures of 98 ^o^C and 65 ^o^C, for this buffer, the actual pH values were about 1.8 and 1 units lower than shown in the Figure for AMOR_GH9A and *Tf*Cel9A, respectively. Accordingly, the apparent gaps in pH-dependency curves are in fact nonexistent. Error bars indicate standard deviations between triplicates.

The optimal operating conditions of AMOR_GH9A and *Tf*Cel9A were assessed using carboxymethyl cellulose (CMC) as a model substrate (Fig 3). Of note, despite the large amount of data published on *Tf*Cel9A, the pH and temperature dependency of the full-length enzyme have not been addressed in detail before.

The results indicate that *Tf*Cel9A performs best at pH 6.2, 65°C. The pH optimum of AMOR_GH9A is approximately 5.7, while the temperature optimum is 100°C or higher. During the experiment, the highest activity was observed when the boiling point was reached and a further increase in incubation temperature was not possible for practical reasons. After the initial temperature optima assays, all the subsequent AMOR_GH9A reactions with CMC were carried out at 98°C. Note that while the pH of the assay buffers was set at the room temperature, the reported pH optima for both enzymes are hardly affected by the experimental conditions, since the temperature-dependency of the citrate-phosphate buffer is extremely low [36].

The pronounced difference between the temperature optima of AMOR_GH9A and *Tf*Cel9A makes sense when considering the origin of the enzymes. *Thermobifida fusca* is a soil bacterium typically found in decomposing organic matter (e.g. compost or rotting hay), which can heat up to approximately 70°C due to exothermic reactions [38]. In comparison, the temperatures at the Jan Mayen vent field can rise up to 260°C [17] with steep thermal gradients.

In a recent review, Escuder-Rodríguez et al. [8] summarized temperature optima of 185 thermophilic cellulases (64 endoglucanases, 121 exoglucanases) of bacterial and fungal origin. Only six of the listed thermophilic cellulases (3.2%) across all the GH families possess a temperature optimum similar to the optimum of AMOR_GH9A (i.e., ≥100°C). There are six GH9 enzymes in the dataset and AMOR_GH9A has a higher optimal temperature than all of these. Of note, the majority of GH9 cellulases reported in the review (five out of six proteins) seem to be only moderately thermophilic since their temperature optima do not exceed 70°C. The most thermophilic of the six GH9s is CelA cellulase from *Caldicellulosiruptor bescii* [39] with a reported temperature optimum of 95°C (note that this is a multimodular enzyme, also containing a GH48 domain). Although these comparisons have limitations (e.g. due to variation in the conditions used), it is clear that AMOR_GH9A belongs to the most thermophilic cellulases described so far.

CMC assays at pH 5.7 and 98 ^o^C showed that the activity of AMOR_GH9A was almost insensitive to salt. The highest activity was obtained in reactions without added NaCl. The increase of salt concentration gradually reduced activity but even at 2 M NaCl, the remaining activity was still approximately 85% of the base level (0 M NaCl) (S2 Fig). These findings are in a strong contrast with the results obtained for two other enzymes (a thermostable xylanase, AMOR_GH10A, and a thermostable alginate lyase, AMOR_PL7A) recently discovered using the same metagenomic dataset [21, 22]. Unlike AMOR_GH9A, AMOR_GH10A is a salt-dependent enzyme showing low activity at 0 mM NaCl. AMOR_PL7A is less responsive to salt, but requires the addition of ~430 mM NaCl to the buffer to manifest full activity.

### Thermal stability

**Fig 4:**
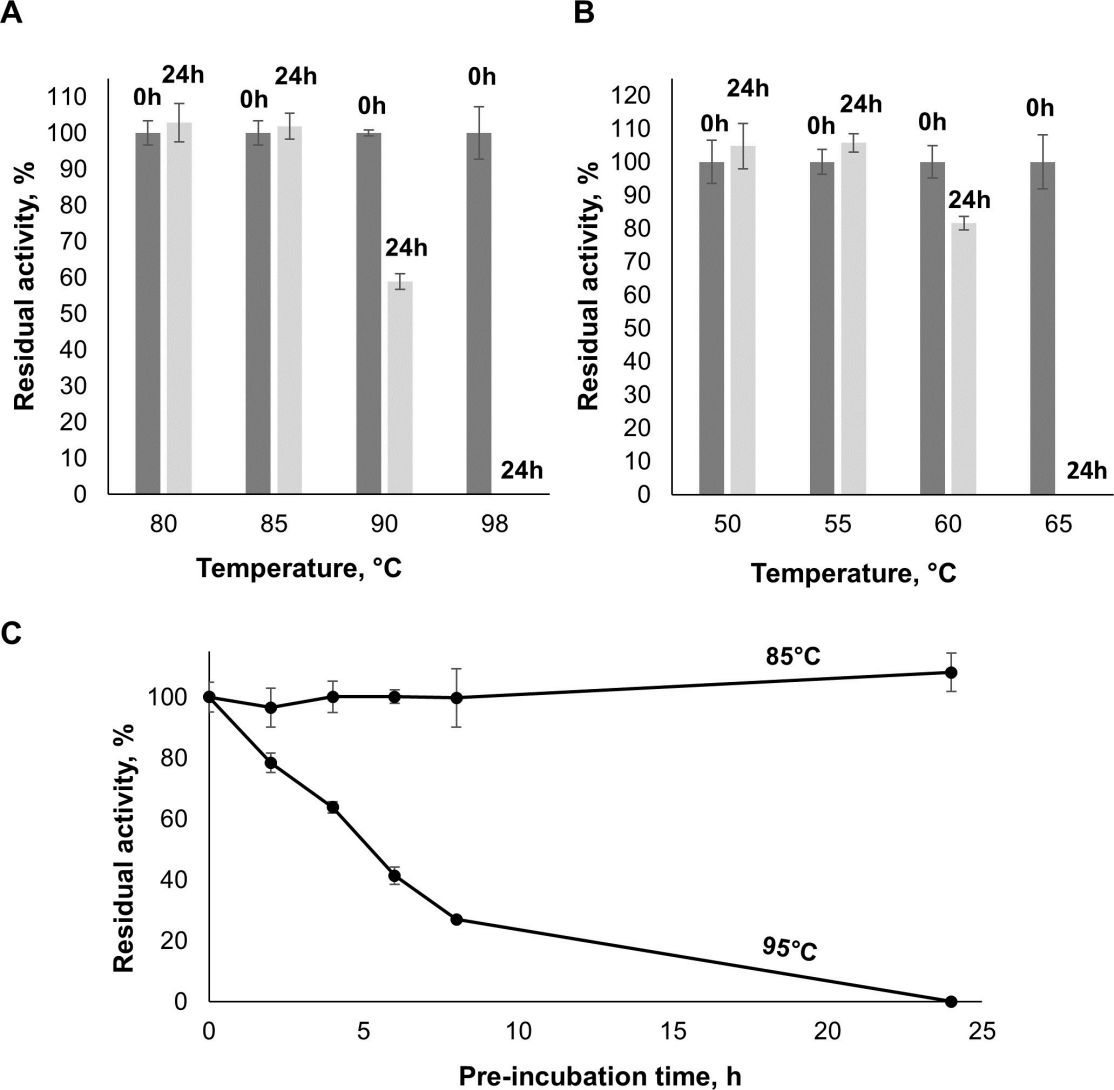
Thermal stability of AMOR_GH9A. Panels (A), (B) and (C) show the residual activity of AMOR_GH9A (A, C) and *Tf*Cel9A (B) in 6 min reactions with CMC after pre-incubation at various temperatures for various time periods (0 or 24 h in panels A and B; multiple time points in panel C). The pre-incubations were done in citrate-phosphate buffer, pH 5.7, or in citrate-phosphate buffer, pH 6.2, for AMOR_GH9A and *Tf*Cel9A, respectively. The activity assays were done at 98 ^o^C or 65 ^o^C and at pH 5.7 or 6.2 for AMOR_GH9A and *Tf*Cel9A, respectively. Error bars indicate standard deviations between triplicates.

**Fig 5:**
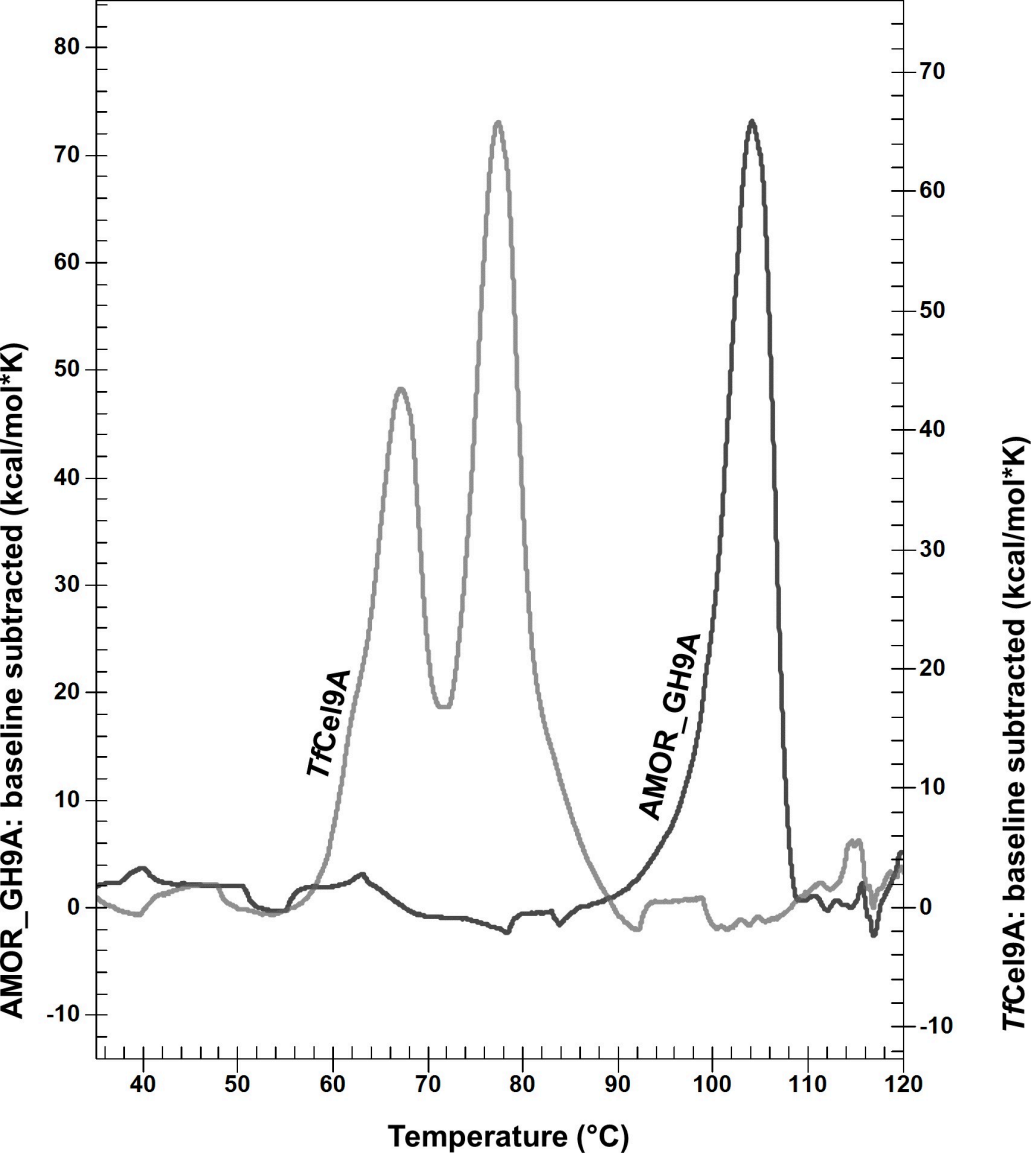
Differential scanning calorimetry (DSC) melting curves for AMOR_GH9A and *Tf*Cel9A. The molar heat capacity of the enzyme solutions is plotted as a function of the temperature. The enzymes were dissolved at 0.5 mg/ml concentration in citrate-phosphate buffer pH 5.7 or citrate-phosphate buffer pH 6.2 for AMOR_GH9A and *Tf*Cel9A, respectively. Before plotting, baseline curves (i.e., buffer only) were subtracted from the protein curves. The heating rate was 1°C per minute. In both cases, the unfolding was irreversible.

The thermal stability of AMOR_GH9A and *Tf*Cel9A was assessed and compared by measuring residual activity on CMC after pre-incubation of the enzymes in citrate-phosphate buffer at optimal pH and various temperatures (Fig 4). AMOR_GH9A and *Tf*Cel9A retained 100% activity after 24 hours of pre-incubation at 85°C and 55°C, respectively. At higher temperatures, the proteins became unstable. It is worth noting that AMOR_GH9A remains active for quite a long time under extreme conditions. For example, our results indicate that the enzyme retains 64% of its activity after 4 hours of pre-incubation at 95°C (Fig 4C). To the best of our knowledge, such degree of thermostability is unparalleled among GH9 cellulases reported so far. Melting curves for AMOR_GH9A and *Tf*Cel9A were obtained using differential scanning calorimetry (DSC). Both AMOR_GH9A and *Tf*Cel9A displayed irreversible unfolding. The melting curve for AMOR_GH9A showed a single peak at approximately 105°C while *Tf*Cel9A demonstrated a two-phase transition with peaks around 65°C and 78°C (Fig 5). A bi-phasic nature of *Tf*Cel9A unfolding is not surprising, considering the complex domain structure of the enzyme (Fig 1). Interestingly, the first *Tf*Cel9A unfolding phase happened at the temperature where the enzyme starts losing its activity (~65°C; Figs 3B & 4B). It is thus conceivable that this first phase corresponds to unfolding of the catalytic domain.

### Substrate specificity

**Fig 6:**
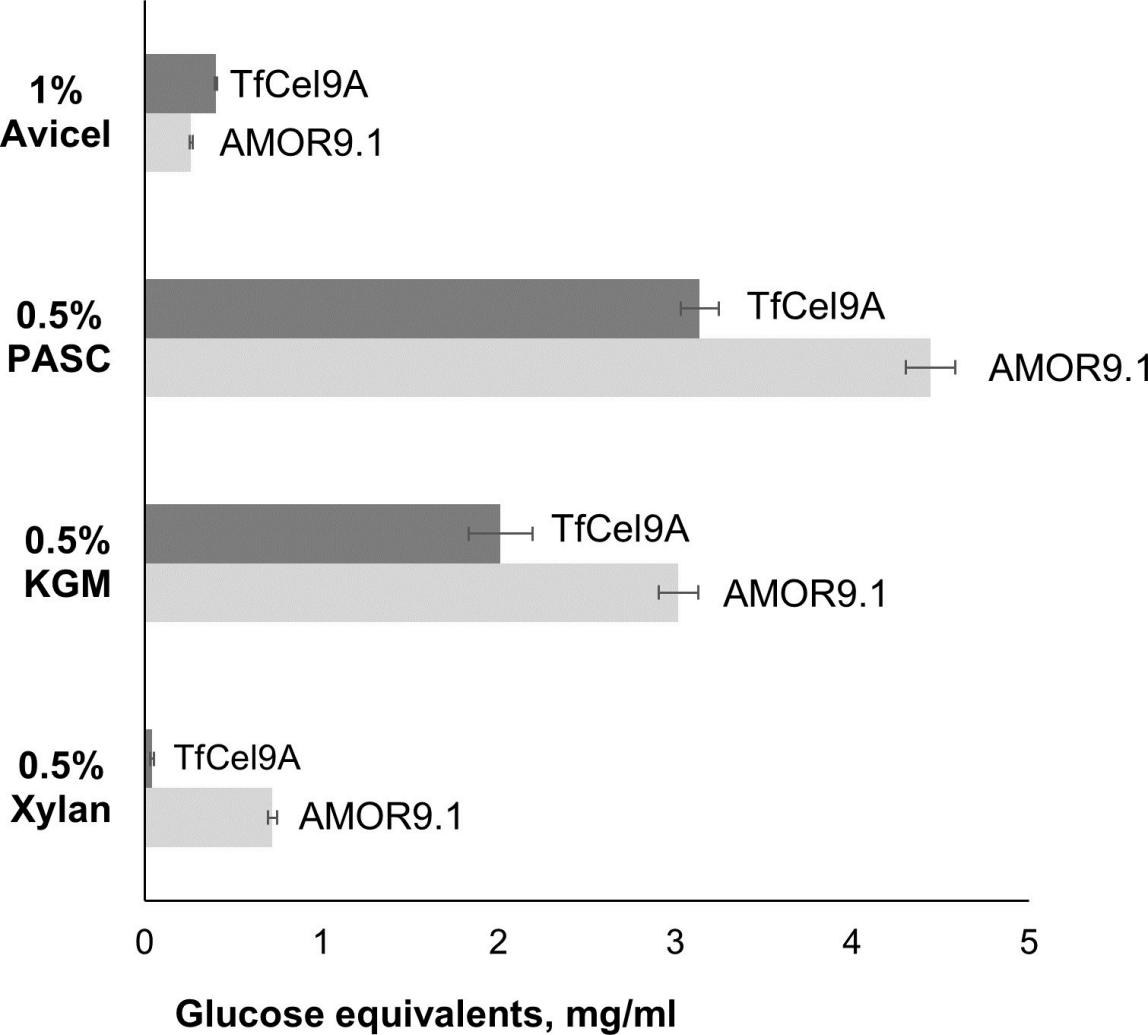
Substrate specificity. The chart shows product release by AMOR_GH9A and *Tf*Cel9A from various substrates after 24h incubation at optimal pH and a temperature not likely to lead to enzyme inactivation. Reaction conditions for AMOR_GH9A: 1 μM enzyme, 85°C, pH 5.7; for *Tf*Cel9A: 1 μM enzyme, 55°C, pH 6.2. The substrate concentration was 1% (w/v) in case of Avicel and 0.5% (w/v) in case of all the other substrates. KGM, konjac glucomannan; xylan, beechwood xylan. Error bars indicate standard deviations between triplicates.

Studies of substrate specificity showed that both AMOR_GH9A and *Tf*Cel9A hydrolyze PASC and Avicel (Fig 6). AMOR_GH9A outperformed *Tf*Cel9A in reactions with amorphous PASC, releasing approximately 1.4 times more glucose equivalents, whereas *Tf*Cel9A showed the highest activity on Avicel. These differences may in part be due to the different architecture and CBM content of the two enzymes (Fig 1). In particular, the only cellulose binding domain of AMOR_GH9A belongs to the subfamily CBM3c, which has relatively weak affinity towards crystalline substrates [32]. In case of *Tf*Cel9A binding to Avicel is likely to be enhanced by the additional C-terminal CBM2 domain. Of note, the performance of both cellulases on Avicel is relatively poor given the enzyme load of 100 nmol per gram of substrate. The reducing end concentration obtained after 24h incubation with *Tf*Cel9A (Fig 6) corresponds to approximately 5% substrate solubilization. Previous studies of the degradation of Avicel by *Tf*Cel9A gave similar results [40].

**Fig 7:**
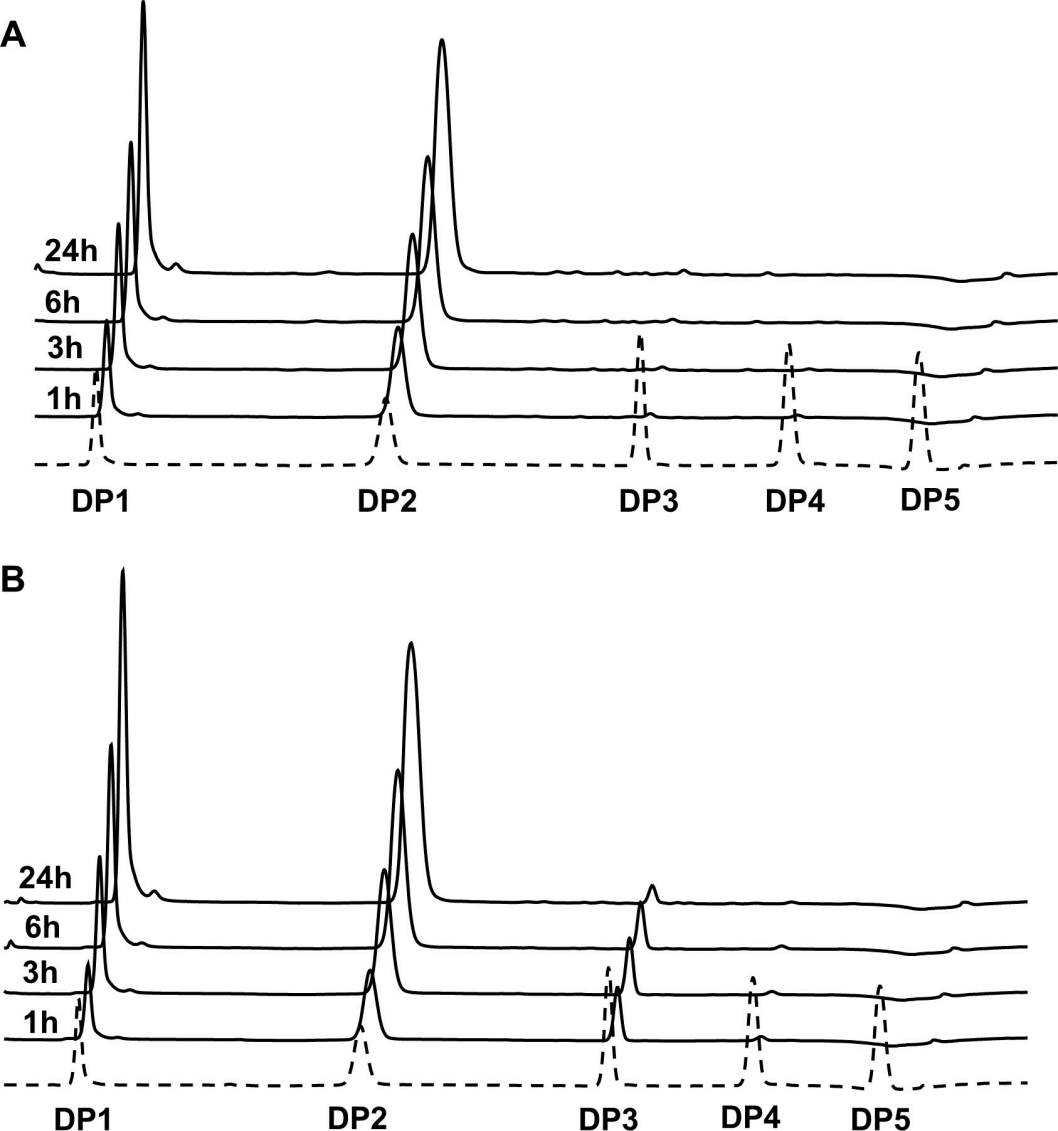
Products generated from Avicel. Panels A and B show cello-oligosaccharides (HPAEC-PAD chromatograms) generated over time from 1% (w/v) Avicel by AMOR_GH9A (A) and *Tf*Cel9A (B). Reaction conditions for AMOR_GH9A: 1 μM enzyme, 85°C, pH 5.7; for TfCel9A: 1 μM enzyme, 55°C, pH 6.2. The dotted line is a chromatogram of a standard sample containing cello-oligosaccharides in the DP1 to DP5 range, each at 100 μM concentration.

The HPAEC-PAD analysis of Avicel depolymerization products revealed some interesting features. Firstly, during the initial phase of the reaction, *Tf*Cel9A generated a significant amount of cellotriose and this trisaccharide was still detectable after 24 hours (Fig 7). The fact that AMOR_GH9A only produced disaccharides and monosaccharides suggests that the two enzymes have different substrate-binding abilities, with *Tf*Cel9A being less capable of cleaving short cello-oligosaccharides such as cellotriose. Indeed, the low ability of *Tf*Cel9A to cleave short oligosaccharides has been observed previously [41]. A second interesting feature is the high level of monosaccharides that are formed. Although disaccharide/monosaccharide ratios need to be used with caution [42], they give an indication of enzyme processivity and the relatively low disaccharide/monosaccharide ratios observed here indicate that the two GH9s are not particularly processive. While some degree of processivity cannot be excluded [33, 41], it may not be a dominating feature of these enzymes.

**Fig 8:**
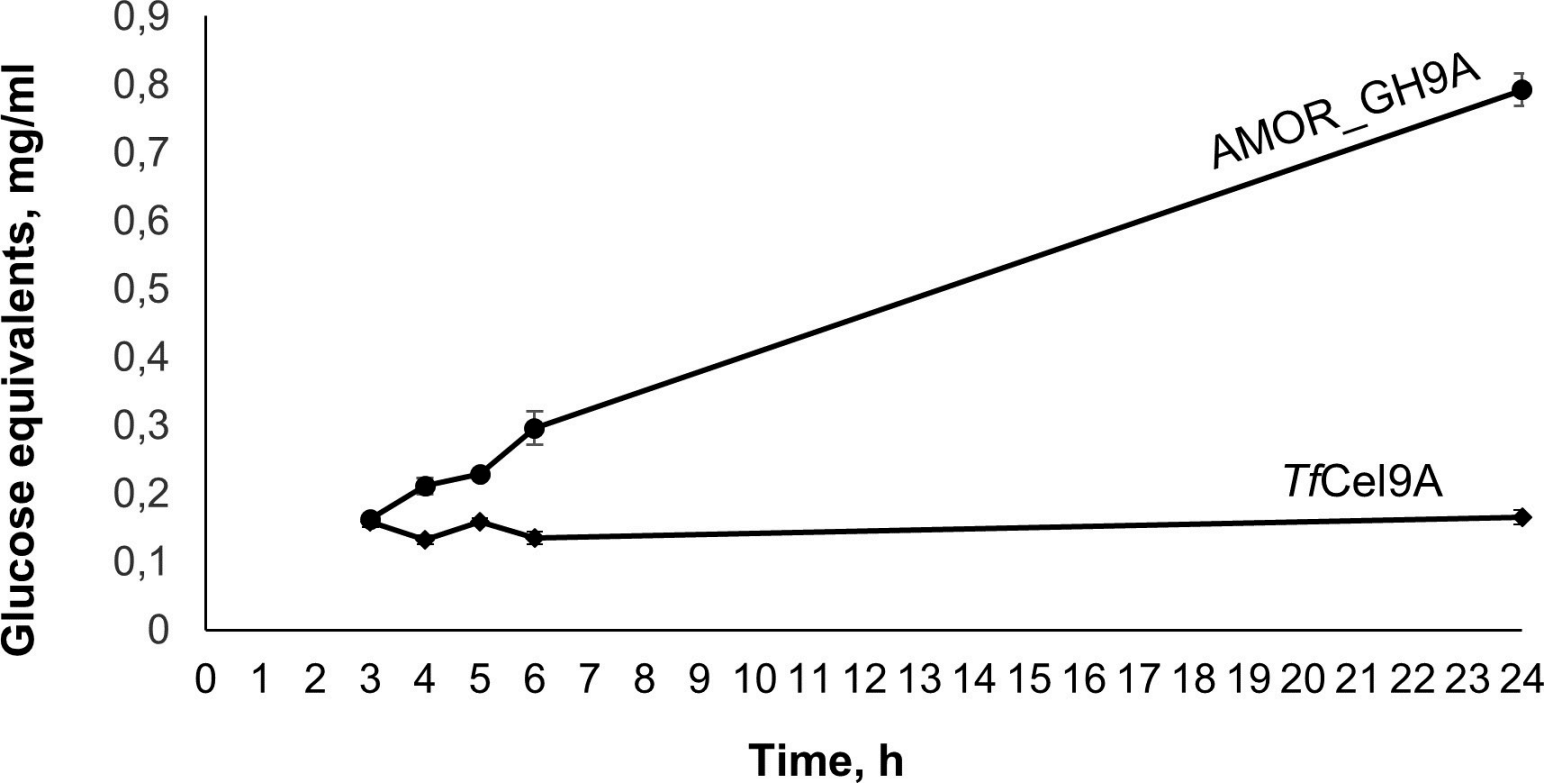
AMOR_GH9A activity on xylan. The chart shows product release by AMOR_GH9A and *Tf*Cel9A from 0.5% (w/v) beechwood xylan at various timepoints. Reaction conditions for AMOR_GH9A: 1 μM enzyme, 85°C, pH 5.7; for TfCel9A: 1 μM enzyme, 55°C, pH 6.2. Error bars indicate standard deviations between triplicates.

**Fig 9:**
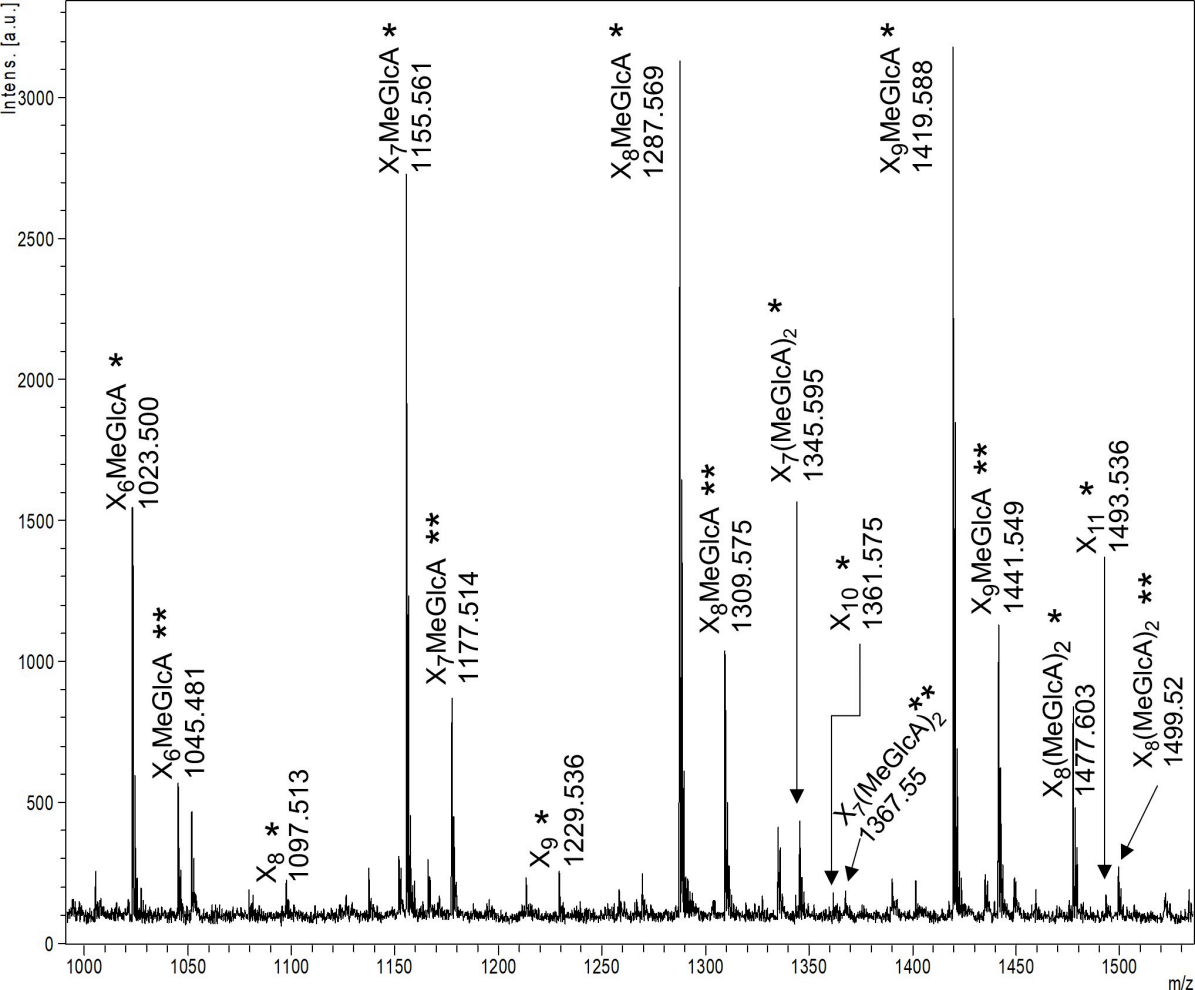
MALDI-TOF MS analysis of products generated from beechwood xylan by AMOR_GH9A. The picture shows a part of a MALDI-TOF spectrum with signals from non-substituted and substituted xylo-oligosaccharides released from 0.5% (w/v) xylan after a 24 hour incubation. Reaction conditions: 1 μM enzyme, 85°C, pH 5.7. X, xylose; MeGlcA, 4-O-methylglucuronic acid; *, sodium adduct; **, sodium salt of a sodium adduct. None of the labeled peaks were observed in the negative control (i.e. a reaction without added enzyme).

**Fig 10:**
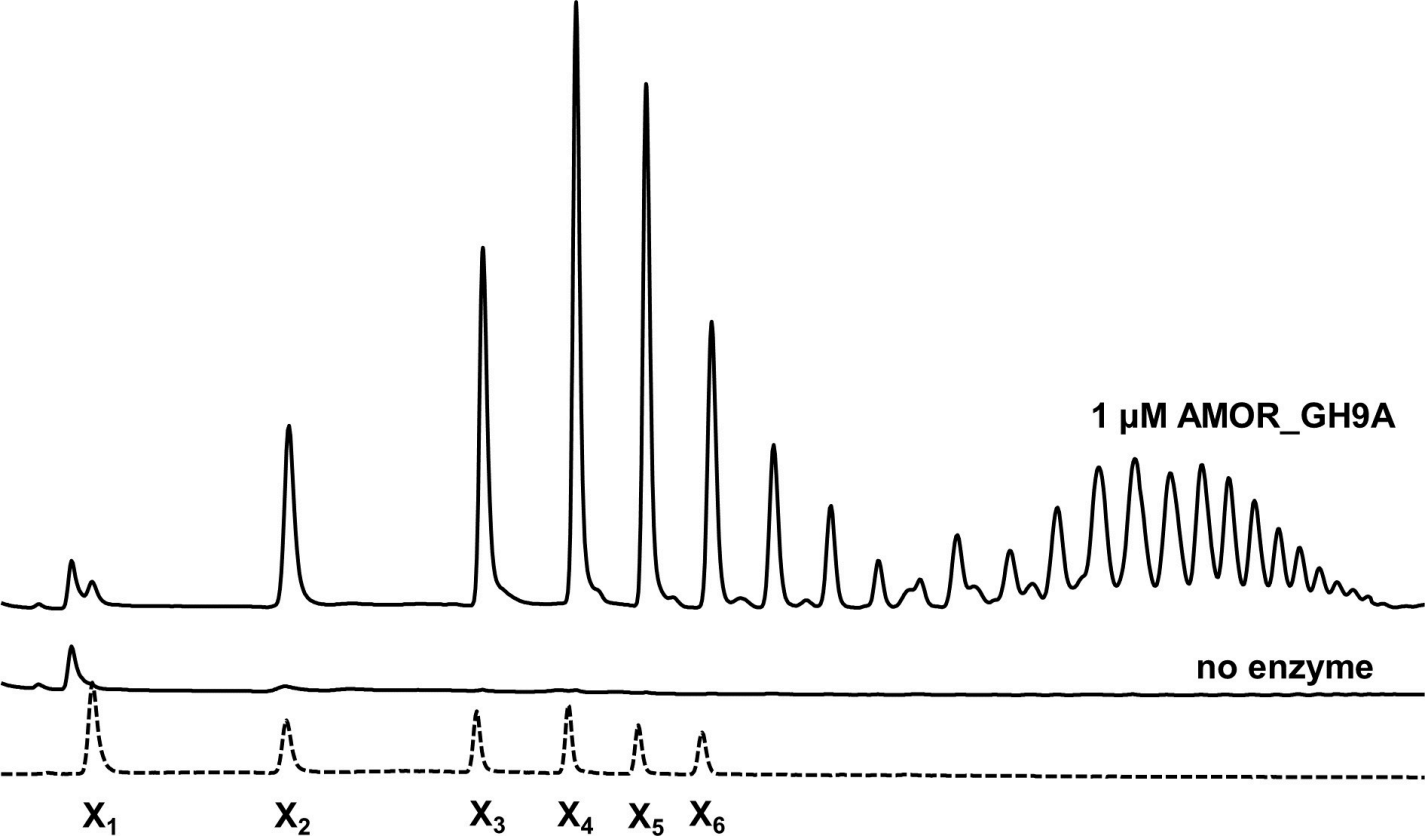
HPAEC-PAD analysis of products generated from beechwood xylan by AMOR_GH9A. The figure shows HPAEC-PAD chromatograms of xylo-oligosaccharides generated after incubation of 0.5% (w/v) beechwood xylan by 1 μM AMOR_GH9A at 85°C, pH 5.7, for 24h. The dotted line is a chromatogram of a standard sample containing xylo-oligosaccharides in the DP1 to DP6 range, each at 0.01 mg/ml concentration. The peaks in the right half of the chromatogram of the reaction sample represent xylo-oligosaccharides with high degree of polymerization (including substituted xylo-oligosaccharides). The “no enzyme” line is a chromatogram of a negative control sample.

The vast majority of characterized GH9 enzymes are cellulases [43]. However, some of these cellulose-targeting enzymes are known to display side activities towards hemicellulosic substrates including glucomannan [44], xylan [45] and xyloglucan [46]. Indeed, we found that both AMOR_GH9A and *Tf*Cel9A are able to hydrolyze konjac glucomannan (Fig 6). Interestingly, AMOR_GH9A showed a clear activity on xylan, in contrast to *Tf*Cel9A (Figs 6 and 8). The ability to hydrolyze xylan is a desirable property considering the high xylan content of several industrially relevant types of plant biomass [47]. MALDI-TOF MS analysis of products released from beechwood xylan showed that AMOR_GH9A generates a mixture of non-substituted xylo-oligosaccharides and xylo-oligosaccharides substituted with methylated glucuronic acid (Fig 9). Chromatographic analysis of products generated from beechwood xylan confirmed that AMOR_GH9A releases a wide variety of substituted and non-substituted xylo-oligosaccharides, including xylobiose and trace amounts of xylose (Fig 10).

### Concluding remarks

*In silico* mining of a metagenomic dataset originating from the Jan Mayen hydrothermal vent field led to the identification of the novel GH9 cellulase named AMOR_GH9, which is among the most thermostable and thermoactive cellulases ever described. The enzyme comprises an N-terminal catalytic domain followed by a CBM3 cellulose binding module and is easy to produce in *E*. *coli*. AMOR_GH9A possesses a remarkably high temperature optimum (≥100°C) and retains 64% of its activity after 4 hours of incubation at 95°C. Direct functional comparison with its closest characterized homolog (*Tf*Cel9A from the model thermophilic bacteria *Thermobifida fusca)* revealed that AMOR_GH9A possesses broader substrate specificity and higher activity on soluble and amorphous substrates (PASC, KGM). Thus, the novel GH9 cellulase demonstrates a set of industrially relevant properties and has the potential to become part of the enzymatic toolbox for biomass conversion.

## Supporting information

S1 Table Primers used for amplification and ligation-independent cloning of the genes encoding AMOR_GH9A and *Tf*Cel9A. Vector complimentary sequences are underlined.
(PDF)

S1 Fig SDS-PAGE of AMOR_GH9A and *Tf*Cel9A cellulases after purification. The predicted MW of AMOR_GH9A and TfCel9A is 73.2 kDa and 91.4 kDa respectively. The sample loading was 5μg for each protein. Note that there is some heterogeneity in the AMOR_GH9A band. We were not able to remove this heterogeneity by additional purification steps or extended boiling of the samples. Note that AMOR_GH9A and *Tf*Cel9A were produced and purified exactly in the same way.
(TIF)

S2 Fig The effect of salt on the activity of AMOR_GH9A. The graph shows product formation after a 6 min reaction with CMC at 98°C in citrate-phosphate buffer, pH 5.7, supplied with NaCl at different concentrations. Error bars indicate standard deviations between triplicates.
(TIF)
