# Supplementary material for: Identification and characterization of a hyperthermophilic GH9 cellulase from the Arctic Mid-Ocean Ridge vent field
Source: PLoS One. 2019 Sep 6;14(9):e0222216. doi: 10.1371/journal.pone.0222216 (PMC6731012; doi:10.1371/journal.pone.0222216)
Supplement: S1 Table — Vector complimentary sequences are underlined. (PDF) [file pone.0222216.s001.pdf]

**Table S1. Primers used for amplification and ligation-independent cloning of the genes encoding AMOR_GH9A and *Tf*Cel9A.** Vector complimentary sequences are underlined.

| **Primer name** | **Primer sequence** |
|---|---|
| AMOR_GH9A-forward (cloning) | TTAAGAAGGAGATATACTATGGCTCCGAAAACCCCGGAAC |
| AMOR_GH9A-reverse (cloning) | AATGGTGGTGATGATGGTGCGCCTGGCTGTTGTGTTGTTTACCTGC |
| *Tf*Cel9A-forward (cloning) | TTAAGAAGGAGATATACTATGGAGCCGGCGTTCAACTATGC |
| *Tf*Cel9A-reverse (cloning) | AATGGTGGTGATGATGGTGCGCCAGCGCGCACGGT |
| pNIC-seq-F (colony PCR) | TGTGAGCGGATAACAATTCC |
| pNIC-seq-R (colony PCR) | AGCAGCCAACTCAGCTTCC |
